# ATAD1 Regulates Neuronal Development and Synapse Formation Through Tuning Mitochondrial Function

**DOI:** 10.3390/ijms26010044

**Published:** 2024-12-24

**Authors:** Hao-Hao Yan, Jia-Jia He, Chuanhai Fu, Jia-Hui Chen, Ai-Hui Tang

**Affiliations:** 1 Hefei National Laboratory for Physical Sciences at the Microscale, MOE Key Laboratory for Membrane-Less Organelles & Cellular Dynamics, Division of Life Sciences and Medicine, University of Science and Technology of China, Hefei 230026, China; yanhaoh@mail.ustc.edu.cn (H.-H.Y.); hejiajia@ustc.edu.cn (J.-J.H.); chuanhai@ustc.edu.cn (C.F.); 2 Anhui Province Key Laboratory of Biomedical Imaging and Intelligent Processing, Institute of Artificial Intelligence, Hefei Comprehensive National Science Center, Hefei 230088, China; 3 Neurodegenerative Disorder Research Center and Biomedical Sciences and Health Laboratory of Anhui Province, University of Science and Technology of China, Hefei 230027, China; 4 Department of Anatomy, School of Basic Medicine, Anhui Medical University, Hefei 230032, China

**Keywords:** ATAD1, neuronal development, synapse formation, mitochondrial dysfunction

## Abstract

Mitochondrial function is essential for synaptic function. ATAD1, an AAA+ protease involved in mitochondrial quality control, governs fission–fusion dynamics within the organelle. However, the distribution and functional role of ATAD1 in neurons remain poorly understood. In this study, we demonstrate that ATAD1 is primarily localized to mitochondria in dendrites and, to a lesser extent, in spines in cultured hippocampal neurons. We found that ATAD1 deficiency disrupts the mitochondrial fission–fusion balance, resulting in mitochondrial fragmentation. This deficiency also impairs dendritic branching, hinders dendritic spine maturation, and reduces glutamatergic synaptic transmission in hippocampal neuron. To further investigate the underlying mechanism, we employed an ATP hydrolysis-deficient mutant of ATAD1 to rescue the neuronal deficits associated with ATAD1 loss. We discovered that the synaptic deficits are independent of the mitochondrial morphology changes but rely on its ATP hydrolysis. Furthermore, we show that ATAD1 loss leads to impaired mitochondrial function, including decreased ATP production, impaired membrane potential, and elevated oxidative stress. In conclusion, our results provide evidence that ATAD1 is crucial for maintaining mitochondrial function and regulating neurodevelopment and synaptic function.

## 1. Introduction

Neurons are characterized by their highly polarized structures, which include distinct axonal and dendritic compartments, and are considered energetically demanding postmitotic cells [1]. Mitochondria are among the most abundant organelles in neurons, serving a critical role in the production of adenosine triphosphate (ATP) through oxidative phosphorylation [2]. In addition to energy production, mitochondria are involved in various essential functions, including Ca^2+^ buffering, the generation of reactive oxygen species (ROS), and neurotransmitter synthesis [3,4]. More importantly, mitochondrial dysfunction has been associated with several neurological and psychiatric disorders [5,6] and the progression of neurodegenerative diseases [7,8,9], underlining the critical role of mitochondrial function in maintaining neuronal health. Given the critical role of mitochondria in eukaryotic cells, particularly in neurons, various quality control mechanisms have evolved to address and repair dysfunctional mitochondria in order to maintain their normal function [10,11,12,13,14].

ATPase Family AAA Domain Containing 1 (ATAD1) aligns itself within the AAA^+^ protease family known for its significant involvement in mitochondrial quality control [15,16], and it facilitates the removal of mislocalized tail-anchored (TA) proteins from the outer mitochondrial membrane, thereby ensuring the proper import of mitochondrial proteins [17]. Recent studies have revealed that mutations in the *atad1* gene are associated with a range of neurological phenotypes [17]. Human patients carrying mutations in the *atad1* exon display severe neurological phenotypes, characterized by encephalopathy and stiff baby syndrome and ischemic injury and motor impairments [18,19,20]. The homozygous variants in *atad1* give rise to severe cerebral atrophy, epileptic-like syndromes, and premature mortality during early childhood [19]. While ATAD1 has been reported to impact synaptic plasticity through its interactions with α-amino-3-hydroxy-5-methyl-4-isoxazolepropionic acid (AMPA) receptors [21,22], it has been found to be predominantly located on the outer membrane of mitochondria in other cell types [23]. We have recently revealed that the ATAD1 homolog plays a critical role in preventing excessive mitochondrial fission in yeast by disrupting the mitochondrial fission machinery through regulating the mitochondrial divisome [24], and consistent with this, its deficiency leads to mitochondrial fragmentation in mouse embryonic fibroblasts (MEFs) [23]. Given these findings, a further investigation into the direct role of ATAD1 in modulating synaptic function via its effects on mitochondrial dynamics is warranted.

In this study, we aim to investigate the role of ATAD1 in neuronal development and synaptic function, with a focus on its contribution to hippocampal neuron mitochondrial dynamics and function. We demonstrated that ATAD1 is primarily localized in the mitochondria in rat hippocampal neurons, and its knockdown leads to a shift of mitochondrial dynamics towards fission. Meanwhile, ATAD1 deficiency impaired neuronal development and reduced dendrite complexity and spine density, especially for spines of mature shape. In addition, a loss of ATAD1 leads to reduced excitatory synaptic formation, as evidenced by reduced PSD-95 density and reduced synaptic transmission. The mutant replacement experiments confirmed that the ATP-hydrolyzing site of ATAD1, which is not required for promoting mitochondrial fusion, is critical for spine formation. Importantly, ATAD1 deficiency impaired mitochondrial function, which is characterized by decreased membrane potential, increased oxidative stress, and decreased ATP production. Together, these findings highlight the critical role of ATAD1 in regulating hippocampal synapse development through modulating mitochondrial functions.

## 2. Results

### 2.1. ATAD1 Maintains the Balance of Mitochondrial Fission and Fusion in Rat Hippocampal Neurons

**Figure 1:**
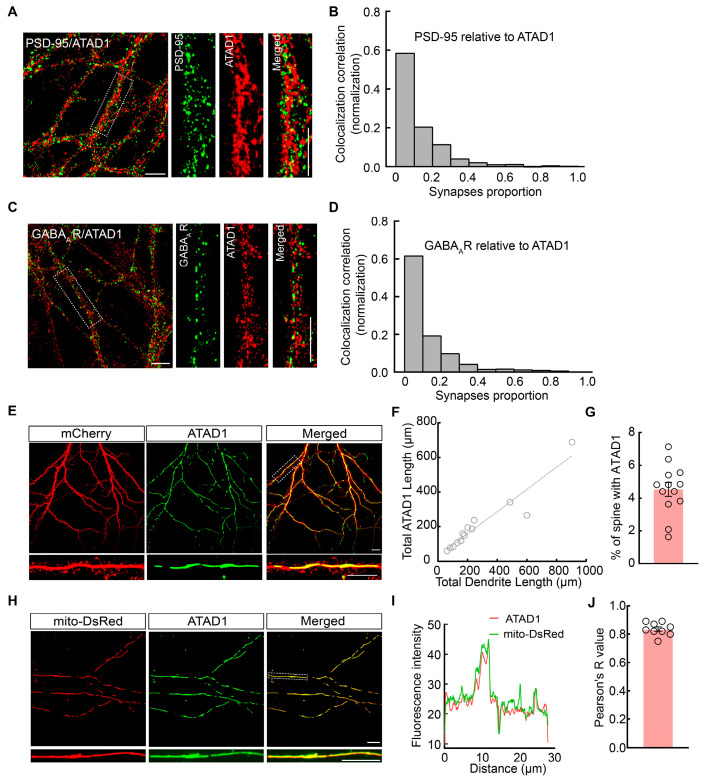
ATAD1 colocalizes with mitochondria. (**A**) Representative images of PSD-95 (green) and ATAD1 staining (red). Boxed regions are enlarged in inserts. Scale bar 10 μm. (**B**) Quantification of PSD-95-ATAD1 colocalization using local correlation (*n* = 9 cells/3 cultures). (**C**) Representative images of GABA_A_R (green) and ATAD1 staining (red). Boxed regions are enlarged in inserts. Scale bar 10 μm. (**D**) Quantification of GABA_A_R-ATAD1 colocalization using local correlation (*n* = 4 cells/2 cultures). (**E**) Representative images of neuron morphology and ATAD1 distribution in cultured rat hippocampal neurons co-expressing oe-ATAD1 (green) and mCherry (red). Boxed regions are enlarged in inserts. Scale bar 10 μm. (**F**) Correlation between ATAD1 length and dendrite length (*n* = 13 cells/3 cultures). (**G**) Quantification of the percent of ATAD1-containing spines along dendrites (*n* = 13 cells/3 cultures). (**H**) Representative colocalization of ATAD1 (green) and mitochondrial (red) in cultured neurons co-expressing oe-ATAD1 and mito-Dsred. Boxed regions are enlarged in inserts. Scale bar 10 μm. (**I**) Colocalization of ATAD1 and mito-Dsred in cultured rat hippocampal neurons. (**J**) Pearson coefficient of ATAD1 and mito-Dsred (*n* = 9 cells/3 cultures).

Previous studies have established that Msp1, an evolutionarily conserved mitochondrial outer-membrane protein, localizes to the mitochondria in yeast, and a significant fraction of human ATAD1 also shows mitochondrial localization [23]. To explore the distribution of ATAD1 in rat hippocampal neurons, we first employed immunofluorescence staining to examine the colocalization of endogenous ATAD1 with the excitatory synapse marker PSD-95 and the inhibitory synapse maker GABA_A_R. Immunofluorescence staining and imaging of rat hippocampal neurons were taken at days in vitro 18–20 (DIV18–20). Analysis of the local correlation between fluorescent signals [25] from PSD-95/GABA_A_R and ATAD1 revealed a minimal colocalization of endogenous ATAD1 with PSD-95 (Figure 1A,B) or GABA_A_R (Figure 1C,D). To further validate this, we constructed a plasmid encoding human ATAD1 protein (oe-ATAD1) and co-transfected neurons with oe-ATAD1 and mCherry for the visualization of ATAD1 and the morphology of neurons. We found that ATAD1 is primarily localized in the dendritic shafts, with only a minimal fraction (4.54%) detected in spines (Figure 1E–G). Additionally, co-transfection with mito-DsRed plasmids to label mitochondria demonstrated a strong colocalization (83.56%) between ATAD1 and mitochondria (Figure 1H–J), supporting the localization of ATAD1 on mitochondria in hippocampal neurons. These results indicate that ATAD1 predominantly localizes to the mitochondria in hippocampal neurons.

**Figure 2:**
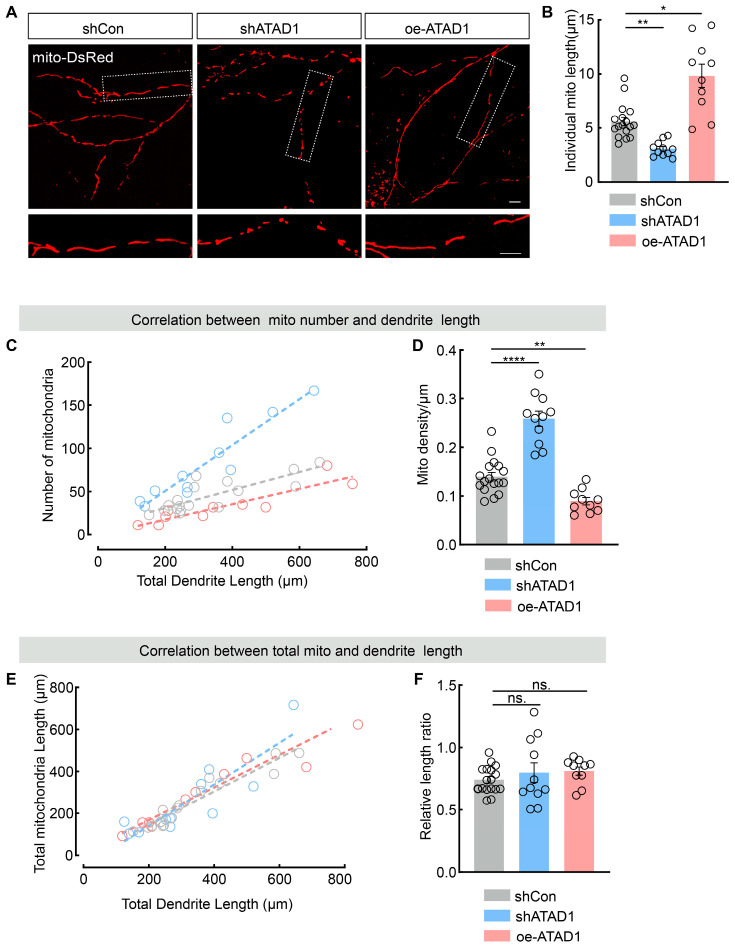
ATAD1 deficiency leads to mitochondrial fragmentation in neurons. (**A**) Representative images of mitochondrial morphology in shCon-expressing, shATAD1-expressing or oe-ATAD1-expressing neurons. Scale bar 5 μm. Boxed regions are enlarged in inserts. Scale bar 5 μm. (**B**) Quantification of individual mitochondrial length along dendrites (*n* = 10–17 cells/3–5 cultures, *p* < 0.0001). (**C**,**D**) The correlation between number of mitochondria and total dendrite length (**C**), and quantification of the mitochondrial density (**D**) (*n* = 10–17 cells/3–5 cultures, *p* < 0.0001) along dendrites. (**E**,**F**) The correlation between total mitochondrial length and total dendrite length (**E**), and relative length ratio (**F**) (*n* = 10–17 cells/3–5 cultures, *p* = 0.2797). Significance was assessed by one-way ANOVA measures (**D**), Welch ANOVA (**F**), or Kruskal–Wallis test (**B**). All data are presented as the mean ± s.e.m. * *p* < 0.05, ** *p* < 0.01, **** *p* < 0.0001; ns., not significant.

Our previous study [24], as well as others [23], demonstrates that ATAD1 prevents excessive mitochondrial fission by regulating the mitochondrial divisome in yeast and HeLa cells. We next examined the impact of ATAD1 deficiency on mitochondrial morphology in neurons. We constructed a plasmid encoding ATAD1 shRNA (shATAD1) to reduce the level of ATAD1 protein. We transfected neurons with shATAD1 to assess the efficacy of ATAD1 knockdown and transfected neurons with scrambled shRNA (shCon) as controls. The expression of shATAD1 in neurons resulted in a significant reduction in ATAD1 fluorescence intensity compared to neurons expressing shCon (Figure S1A,B). This reduction confirms efficient suppression of endogenous ATAD1 expression. Co-expression of shATAD1 and mito-DsRed in neurons (Figure 2A) revealed in a significant decrease (by 45.11%) in individual mitochondrial length and a dramatic increase (by 84.97%) in mitochondrial density compared to control neurons expressing shCon and mito-DsRed, indicating that the ATAD1 deficiency shifts the mitochondrial dynamics towards fission in neurons (Figure 2B–D). Conversely, the overexpression of ATAD1 in neurons resulted in a significant increase (by 75.75%) in individual mitochondrial length and a decrease (by 35.92%) in mitochondrial density (Figure 2B–D), indicating a shift towards fusion. To further confirm this, we performed mitochondrial staining using MitoTracker. Consistent with previous results, shATAD1-expressing neurons exhibited shorter mitochondria compared to the control, whereas the oe-ATAD-expressing neurons displayed longer mitochondria than the control (Figure S2A,B). Furthermore, the co-expression of oe-ATAD1 (shATAD1-resistent human ATAD1) rescued the effects of ATAD1 knockdown, restoring mitochondrial fusion and recovering individual mitochondrial length and density to control levels (Figure S3A–D). However, there were no observable changes in the total length of neuronal mitochondria (Figure 2E,F). These data together suggest that the suppression of endogenous ATAD1 does not alter the total length of neuronal mitochondria but primarily influences mitochondrial dynamics by modulating mitochondrial fission. Collectively, these findings support the notion that ATAD1 controls the balance of mitochondrial fission and fusion in neurons.

### 2.2. Loss of ATAD1 Impaired the Development of Neuron

**Figure 3:**
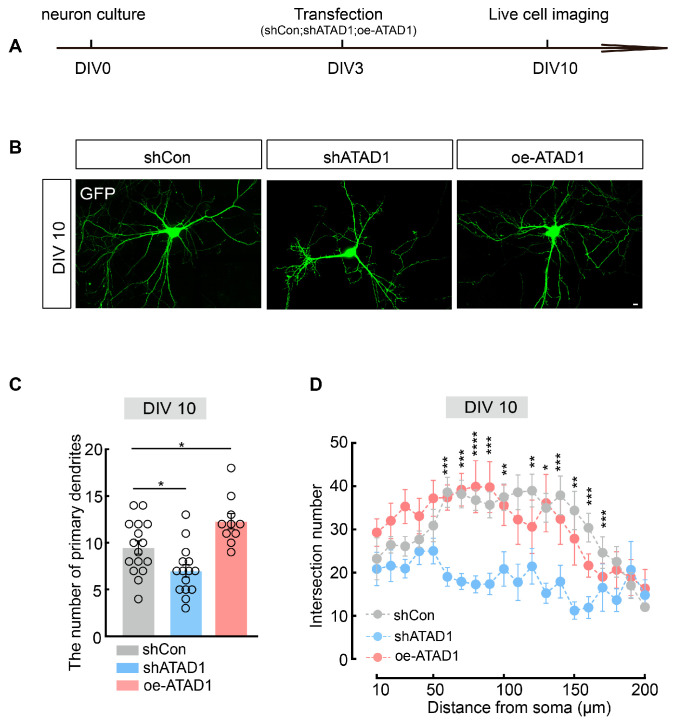
ATAD1 deficiency leads to impaired neurodevelopment in cultured hippocampal neurons. (**A**) Schematic timeline for transfection experimental design of cultured rat hippocampal neurons. (**B**) Representative images of neuronal morphology and dendritic branching in shCon-expressing, shATAD1-expressing, or oe-ATAD1-expressing neurons in DIV10. Scale bar 100 μm. (**C**,**D**) Quantification of primary dendrites number and dendritic intersection number in neurons expressing shCon, shATAD1 or oe-ATAD1 at DIV10 (*n* = 10–16 cells/3 cultures, (**C**), *p* = 0.001, (**D**), *p* = 0.001). Significance was assessed by one-way ANOVA measures (**C**), or two-way ANOVA measures (**D**). All data are presented as the mean ± s.e.m. * *p* < 0.05, ** *p* < 0.01, *** *p* < 0.001; **** *p* < 0.0001.

Previous research has indicated that variants of ATAD1 can lead to impaired synaptic transmission, as well as deficits in memory and social behavior [22]. Moreover, the absence of ATAD1 in mouse models is associated with a reduction in the volumes of both the hippocampus and cortex, along with the manifestation of abnormal activity patterns in the open field activity test [26]. However, the effects of ATAD1 on neuronal development remain unclear. Therefore, we asked whether loss of ATAD1 influences the neuronal development. To test this, neurons were transfected with plasmids encoding shATAD1, oe-ATAD1 or shCon at DIV3 and imaged at DIV10 (Figure 3A). The number of primary dendrites and dendritic complexity (quantified by counting the intersections between dendritic branches and concentric circles of increasing radius in Sholl analysis) were significantly decreased in shATAD1-expressing neurons at DIV10 compared with control neurons (Figure 3B–D). Conversely, neurons with overexpression of ATAD1 maintained the dendritic branching number but showed an increase in the number of primary dendrites at DIV10 (Figure 3B–D). These results demonstrate that ATAD1 is required for the normal branching of neuronal dendrites. 

### 2.3. ATAD1 Deficiency Leads to Decreased Spine Density and Alterations in Dendritic Spine Morphology

**Figure 4:**
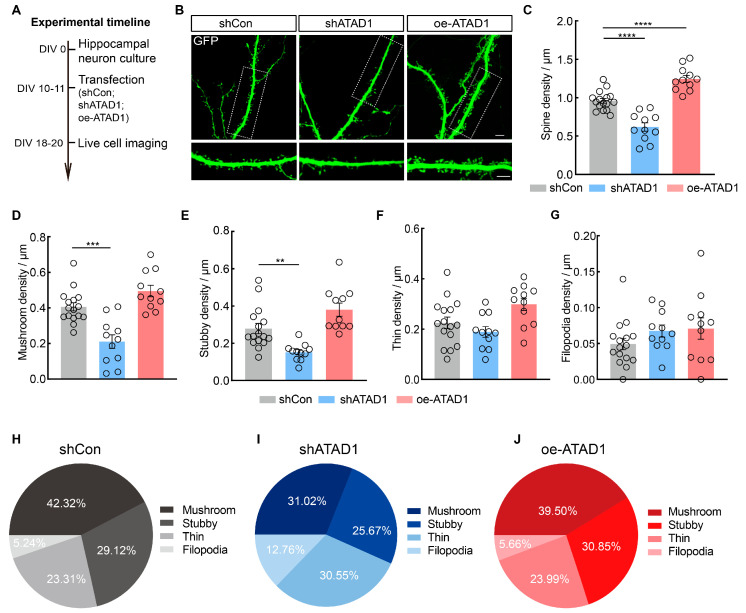
ATAD1 knockdown reduces neuronal maturity in cultured hippocampal neurons. (**A**) Schematic timeline for transfection experimental design in cultured rat hippocampal neurons. (**B**) Representative images of neuronal morphology and dendritic spines in cultured rat hippocampal neurons expressing shCon, shATAD1 or oe-ATAD1. Scale bar 5 μm. Boxed regions are enlarged in inserts. Scale bar 5 μm. (**C**) Quantification of neuronal dendritic spines density in cultured rat hippocampal neurons expressing shCon, shATAD1 or oe-ATAD1. (*n* = 11–16 cells/3–5 cultures, *p* < 0.0001). (**D**–**G**) Quantification of mushroom density (**D**), stubby density (**E**), thin density (**F**) and filopodia density (**G**) in cultured rat hippocampal neurons expressing shCon, shATAD1 or oe-ATAD1 (*n* = 11–16 cells/3–5 cultures, (**D**), *p* < 0.0001, (**E**), *p* < 0.0001, (**F**), *p* = 0.0117, (**G**), *p* = 0.2664). (**H**–**J**) Quantification of distribution of spine morphologies in cultured rat hippocampal neurons expressing shCon (**H**), shATAD1 (**I**) or oe-ATAD1 (**J**) (*n* = 11–16 cells/3–5 cultures). Significance was assessed by one-way ANOVA measures (**C**,**D**,**F**,**G**), or Kruskal–Wallis test (**E**). All data are presented as the mean ± s.e.m. ** *p* < 0.01, *** *p* < 0.001; **** *p* < 0.0001.

We next examined whether ATAD1 deficiency regulates dendritic spines. Cultured hippocampal neurons were transfected at DIV10-11 and imaged at DIV18-20 (Figure 4A). Neurons expressing shATAD1 showed a significant reduction (by 35.47%) in the density of dendritic spines (Figure 4B,C) and the effect of ATAD1 knockdown was fully rescued by co-transfection of oe-ATAD1, which restored the density of dendritic spines (Figure S4A,B). To further investigate the impact of ATAD1 on spine morphology, we categorized dendritic spines into four distinct classes: filopodia, stubby, thin and mushroom [27]. We observed that neurons expressing shATAD1 exhibited a significant reduction in the density of mushroom (shCon VS. shATAD1 *** *p* = 0.001) and stubby (shCon VS. shATAD1 ** *p* = 0.0044) spines, but no statistical changes in density of thin (shCon VS. shATAD1 *p* = 0.4583) and filopodia (shCon VS. shATAD1 *p* = 0.3631) spines compared to control neurons (Figure 4D–G). However, neurons expressing oe-ATAD1 showed a significant increase in spine density (shCon VS. oe-ATAD1 **** *p <* 0.0001), especially in density of mushroom (shCon VS. oe-ATAD1 *p* = 0.0801) and stubby spines (shCon VS. oe-ATAD1 *p* = 0.0974), although these differences were not statistically significant (Figure 4B–G). Filopodia are immature projections widely regarded as precursors to dendritic spines, while mushroom spines are considered mature structures [28]. Then we examined the impact of ATAD1 on spine maturation by analyzing the proportion of different spine morphologies. Our results showed that in neurons expressing shATAD1, the percentage of filopodia increased from 5.24% to 12.76% and the proportion of mushroom spines decreased from 42.32% to 31.02% (Figure 4H,I). Neurons expressing oe-ATAD1 showed a similar distribution of different spine morphologies compared to neurons expressing shCon (Figure 4H,J). Our analysis of spine morphology revealed a reduction in the fraction of mushroom-shaped spines and an increase in the fraction of filopodia-shaped spines in the shATAD1-expressing neurons, indicating a dependency of spine maturation on ATAD1. These results collectively indicate that ATAD1 regulates both dendritic spine density and maturation.

### 2.4. ATAD1 Is Essential for Excitatory Synapse Formation and Synaptic Transmission

**Figure 5:**
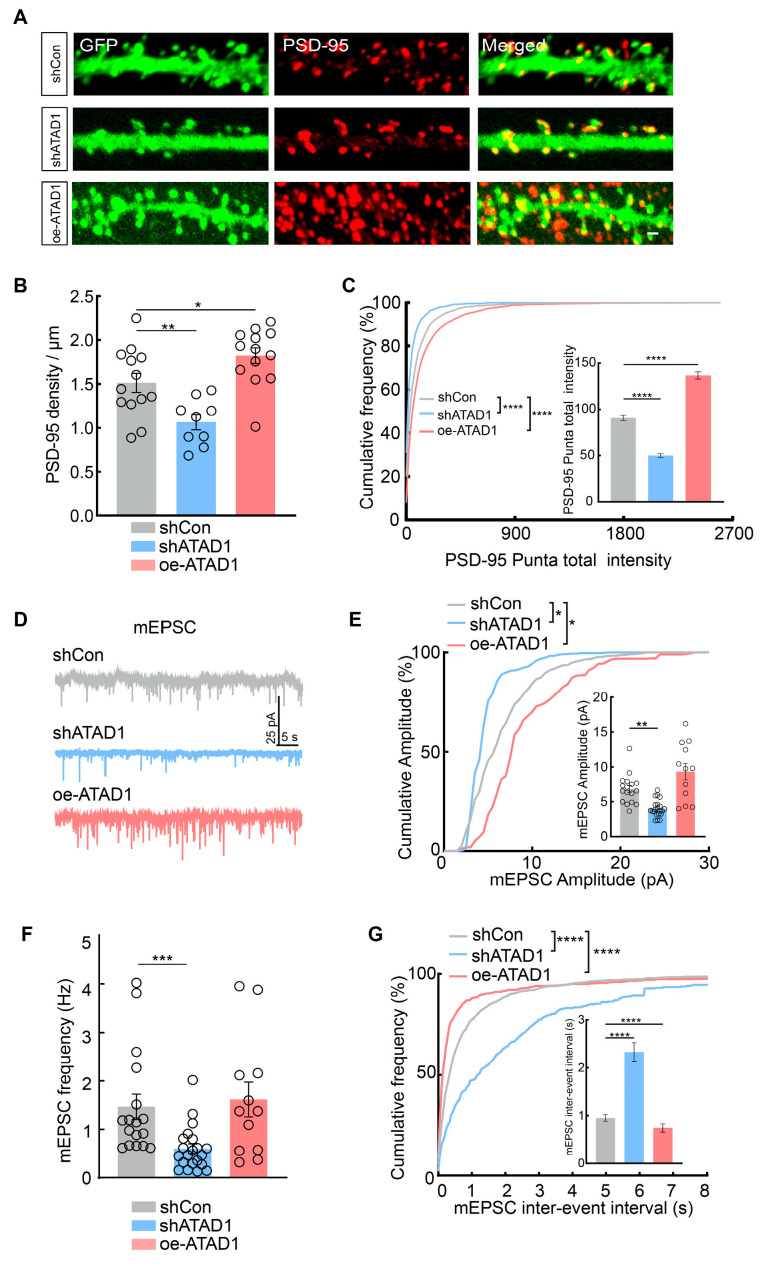
ATAD1 knockdown reduces synaptic transmission in cultured hippocampal neurons. (**A**) Representative images of PSD-95 staining (red) in cultured hippocampal neurons expressing shCon, shATAD1 or oe-ATAD1. Scale bar 1 μm. (**B**) Quantification of PSD-95 density along dendrites in cultured rat hippocampal neurons expressing shCon, shATAD1 or oe-ATAD1 (*n* = 9–13 cells/3 cultures, *p* < 0.0001). (**C**) Quantification of PSD-95 indensity (right) and cumulative probability curve (left) in expressing shCon, shATAD1 or oe-ATAD1 (*n* = 9–13 cells/3 cultures, left, shCon VS. shATAD1 **** *p* < 0.0001, shCon VS. oe-ATAD1, **** *p* < 0.0001, right, *p* < 0.0001). (**D**) Representative traces of mEPSC in neurons expressing shCon, shATAD1 or oe-ATAD1. (**E**) Quantification of mEPSC amplitude (right) and cumulative probability curve (left) in cultured rat hippocampal neurons expressing shCon, shATAD1 or oe-ATAD1 (*n* = 12–21 cells/3 cultures, left, shCon VS. shATAD1, * *p* < 0.0301, shCon VS. oe-ATAD1, * *p* = 0.0497, right, *p* < 0.0001). (**F**) Quantification of mEPSC frequency in cultured rat hippocampal neurons expressing shCon, shATAD1 or oe-ATAD1 (*n* = 12–21 cells/3 cultures, *p* = 0.0004). (**G**) Quantification of mEPSC inter-event interval (right) and cumulative probability curve (left) in cultured rat hippocampal neurons expressing shCon, shATAD1 or oe-ATAD1. (*n* = 12–21 cells/3 cultures, left, shCon VS. shATAD1, **** *p* < 0.0001; shCon VS. oe-ATAD1, **** *p* < 0.0001, right, *p* < 0.0001). Significance was assessed by one-way ANOVA measures (**B**), Kruskal-Wallis test (**C**,**E**–**G**), or K-S test (**C**,**E**,**G**). All data are presented as the mean ± s.e.m. * *p* < 0.05, ** *p* < 0.01, *** *p* < 0.001; **** *p* < 0.0001.

Most excitatory synapses are located on dendritic spines [29]. We used immunofluorescence staining with excitatory synapse marker PSD-95 to examine postsynaptic excitatory synapses in cultured hippocampal neurons. The density and intensity were then estimated by quantifying PSD-95 puncta in neuronal dendrites. Our results revealed a significant 29.57% decrease in PSD-95 density in neurons expressing shATAD1 compared to neurons expressing shCon (Figure 5A,B). Additionally, we quantified the fluorescence intensity of synaptic puncta to represent the level of these postsynaptic proteins and observed a significant decrease in the puncta intensity of PSD-95 in shATAD1 neurons compared to control neurons (Figure 5C). Conversely, overexpression of ATAD1 in neurons resulted in a significant increase in the density and fluorescence intensity of PSD-95 puncta (Figure 5B,C). These results suggest that ATAD1 deficiency leads to defects in the formation of excitatory synapses. The decrease in spine and PSD-95 due to ATAD1 knockdown is expected to result in a decrease in basal synaptic transmission. To examine this, we recorded miniature excitatory postsynaptic currents (mEPSCs) in cultured hippocampal neurons using whole-cell voltage-clamp recordings. We observed a significant decrease in both mEPSC frequency and amplitude in the shATAD1-expressing neurons (Figure 5D,F). Neurons expressing shATAD1 showed a significant increase in the cumulative probability of inter-event intervals (Figure 5G). Conversely, neurons expressing oe-ATAD1 exhibited increases in both mEPSC frequency and amplitude, along with a decrease in inter-event intervals (Figure 5D–G). These results demonstrate that ATAD1 effectively modulates the excitatory synaptic transmission onto the neuron. Taken together, these findings strongly support the pivotal involvement of ATAD1 in shaping excitatory synapse formation and basal glutamatergic synaptic transmission in hippocampal neuron.

### 2.5. The ATP Hydrolysis Site of ATAD1 Is Essential for Spine Formation

**Figure 6:**
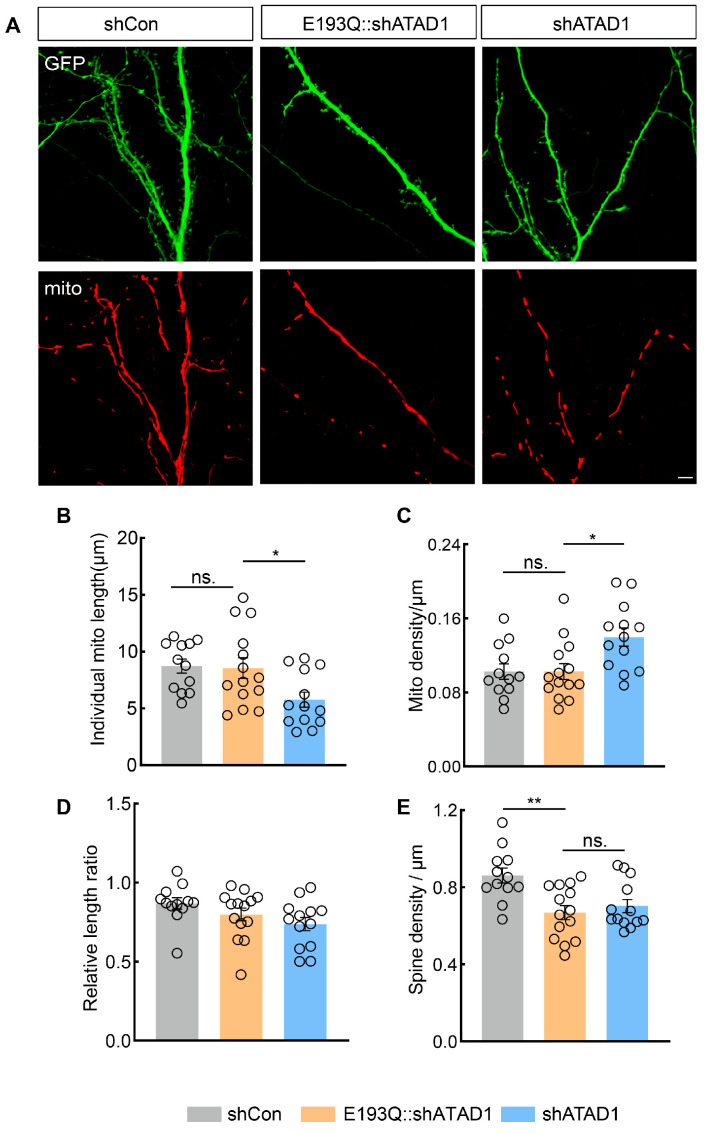
The hydrolysis site mutant of ATAD1 is unable to rescue the reduction in synaptic density. (**A**) Representative images of spine (Green) and mitochondrial morphology (red) in shCon-expressing, E193Q::shATAD1-expressing (E193Q and shATAD1-coexpressing) or shATAD1-expressing neurons. Scale bar 5 μm. (**B**–**D**) Quantification of individual mitochondrial length (**B**), mitochondrial density (**C**) and relative length ratio (**E**) (*n* = 12–14 cells/3 cultures, (**B**), *p* = 0.0154, (**C**), *p* = 0.0017, (**D**), *p* = 0.0887). (**E**) Quantification of neuronal dendritic spines density in cultured rat hippocampal neurons expressing shCon-expressing, E193Q::shATAD1-expressing or shATAD1-expressing (*n* = 12–14 cells/3 cultures, *p* = 0.0018). Significance was assessed by one-way ANOVA measures (**B**–**E**). All data are presented as the mean ± s.e.m. * *p* < 0.05, ** *p* < 0.01, ns., not significant.

To test whether the impact of ATAD1 on spine development is through its effect on mitochondrial fission, we constructed ATAD1 mutant, an ATP hydrolysis-deficient mutant (E193Q), and cotransfected it with shATAD1 to test whether the spine deficit can be rescued. Firstly, we assessed the expression levels of ATAD1 in neurons co-expressing E193Q and shATAD1 using immunostaining. The results showed that the expression levels of ATAD1 in the coexpressing E193Q and shATAD1 neurons were comparable to those in the control (Figure S5A,B). Expression of E193Q fully rescued the decrease in mitochondrial length and the increase in mitochondrial density induced by shATAD1 (Figure 6A–C). Consistent with the findings in shATAD1-expressing neurons and ATAD1-expressing neurons, co-expression of E193Q and shATAD1 did not lead to any changes in the total mitochondrial length (Figure 6D). This is consistent with previous observations on the effect of the same mutants in the homologous Yta4 in yeast [24]. However, E193Q failed in rescuing the decreased spine density induced by shATAD1 (Figure 6A,E). These results demonstrated that the restoration of mitochondrial morphology itself does not lead to a recovery in the density of neuronal spines, and the impact of ATAD1 on spine formation depends on its ATP hydrolysis activity. Therefore, the impairment in spine formation caused by ATAD1 deletion may be attributed to mitochondrial dysfunction resulting from the loss of ATAD1.

### 2.6. Reduced ATAD1 Impaired Mitochondrial Functions

**Figure 7:**
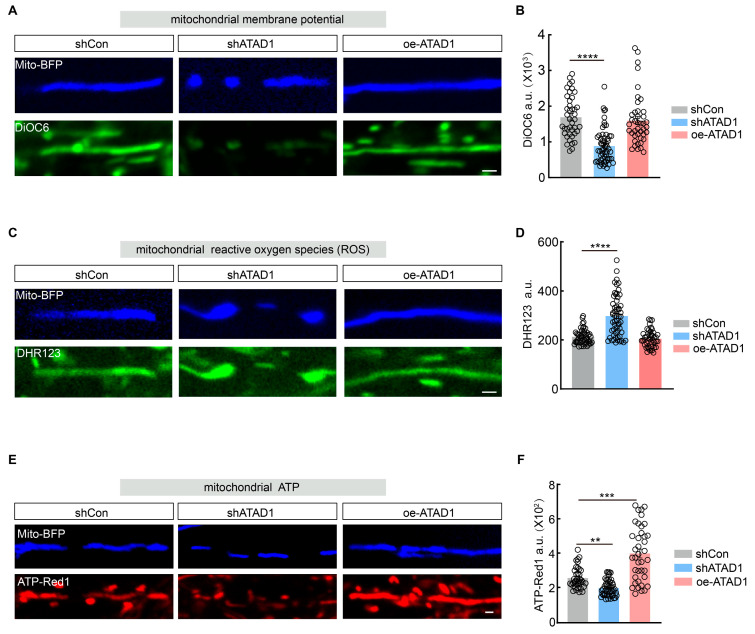
ATAD1 deficiency disrupts mitochondrial function in cultured hippocampal neurons. (**A**) Representative images of DIOC6 staining (green, labeling mitochondrial membrane potential, ΔΨ) in shCon-expressing-control, shATAD1-expressing and oe-ATAD1-expressing neuron. Scale bar 1 μm. (**B**) Quantification of DIOC6 fluorescence intensity in shCon-expressing-control, shATAD1-expressing and oe-ATAD1-expressing neurons (*n* = 41–57 mitochondria /3 cultures, *p* < 0.0001). (**C**) Representative images of DHR123 staining (green, labeling mitochondrial ROS) in shCon-expressing-control, shATAD1-expressing and oe-ATAD1-expressing neurons. Scale bar 1 μm. (**D**) Quantification of DHR123 fluorescence intensity (*n* = 41–57 mitochondria/3 cultures, *p* < 0.0001). (**E**) Representative images of ATP-Red1 staining (red, labeling mitochondrial ATP) in shCon-expressing-control, shATAD1-expressing and oe-ATAD1-expressing neuron. Scale bar 1 μm. (**F**) Quantification of ATP-Red1 fluorescence intensity (*n* = 39–47 mitochondria/3 cultures; *p* < 0.0001). Significance was assessed by Kruskal–Wallis test (**B**,**D**,**F**). All data are presented as the mean ± s.e.m. ** *p* < 0.01, *** *p* < 0.001; **** *p* < 0.0001.

The absence of ATAD1 in mice leads to a loss of mitochondrial proteins in the brain, suggesting its crucial role in maintaining mitochondrial function [17]. Therefore, we examined the impact of ATAD1 knockdown on mitochondrial membrane potential, reactive oxygen species (ROS), ATP homeostasis, and Ca^2+^ signals in neurons. We first utilized a fluorescent probe, DiOC6, to assess mitochondrial membrane potential (ΔΨm) and observed a significant reduction in DiOC6 fluorescence (Figure 7A,B) in neurons expressing shATAD1. Subsequently, we measured changes in ROS using the fluorescent probe DHR123 and revealed an increase in DHR123 fluorescence (Figure 7C,D) in shATAD1-expressing neurons. However, there is no significant difference in DiOC6 and DHR123 fluorescence in oe-ATAD1-expressing neurons (Figure 7A–D). These data indicated a reduced mitochondrial membrane potential and a heightened oxidative stress.

To further explore the role of ATAD1 in ATP homeostasis in neurons, we employed a specialized fluorescent probe called ATP-Red1, which rapidly responds to ATP concentrations within mitochondria. Notably, shATAD1-expressing neurons exhibited a significant decrease in ATP-Red1 fluorescence, whereas oe-ATAD1-expressing neurons showed an increase compared to shCon-expressing neurons (Figure 7E,F). This suggests that ATAD1 positively modulates intracellular mitochondrial ATP. Additionally, we investigated whether ATAD1 deficiency modulates the ability of mitochondria to buffer Ca^2+^. We transfected neurons with Mito-rGECO (a red fluorescent mitochondrial Ca^2+^ sensor) to monitor mitoCa^2+^ and found that neuronal dendritic mitochondria exhibited transient elevations of mitochondrial Ca^2+^ (mCaT) (Figure S6A,B). The co-expression of shATAD1 and Mito-rGECO in neurons resulted in a significant decrease (by 39.99%) in mCaT amplitude (Figure S6C,D). In contrast, the overexpression of ATAD1 did not result in observable changes in either the amplitude or frequency of mCaT compared to control neurons (Figure S6D,E).

These findings demonstrate that ATAD1 deficiency leads to decreased ATP production, impaired mitochondrial membrane potential, and elevated mitochondrial oxidative stress in hippocampal neurons. Given the critical role of these mitochondrial functions in neuronal development, the alterations in these functions may contribute to the significant deficits in dendritic morphology and synapse formation in response to ATAD1 deficiency. 

## 3. Discussion

Mitochondrial function plays a crucial role in neuronal development and synapse formation. Our study reveals a mechanistic link between ATAD1 deficiency and its detrimental effects on mitochondrial function, ultimately affecting neuronal development, dendritic spine maturation, and synaptic transmission. First, we demonstrated that ATAD1 colocalizes with mitochondria and modulates mitochondrial morphology in cultured hippocampal neurons. Secondly, the absence of ATAD1 in cultured hippocampal neurons lead to significant impairments in synaptic transmission accompanied by reduced dendritic branching, dendritic spine maturation and the altered formation of excitatory synapses. Lastly, our research findings indicate that the abnormalities of neuronal spines caused by the loss of ATAD1 are not dependent on mitochondrial fission but rather result from impaired mitochondrial function. Our findings unveil a mechanism by which ATAD1-mediated mitochondrial function modulates synaptic function.

ATAD1 has established its prominence by playing a pivotal regulatory role in maintaining mitochondrial quality on the mitochondrial outer membrane, as evidenced by a series of previous studies [16,23,30,31,32]. Specifically, ATAD1 contributes to mitochondrial homeostasis by orchestrating the removal of mislocalized tail-anchored (TA) proteins from the mitochondrial outer membrane [31,33]. Recent research has unveiled an additional dimension to ATAD1’s functionality by demonstrating that beyond its role in quality control, ATAD1 exerts a significant impact on mitochondrial morphology and intracellular signal pathways. This is exemplified by its direct interaction with mTOR at the mitochondria–lysosome interface, thereby influencing mTOR signaling [34]. Noteworthy observations in *ATAD1-KO* mouse embryonic fibroblasts and ATAD1-knockdown HeLa cells have revealed a distinct alteration in mitochondrial morphology, characterized by fragmentation [23]. Further insights emerge from our recent study showing that ATAD1 interacts with the mitochondrial divisome to inhibit mitochondrial fission [24]. Our finding that the loss of ATAD1 in neurons leads to a shift of the mitochondrial fission–fusion balance towards fission reinforces the notion that ATAD1 has multifaceted role in governing mitochondrial dynamics and functions.

ATAD1 is highly expressed in the brain, particularly in the CA1 region, where it has been shown to function as a neuroprotective factor for neurons. ATAD1 knockout mice exhibit severe neurological deficits, characterized by impairments in learning and memory, ultimately leading to seizure-like conditions [21]. Furthermore, analysis of exome sequencing data from patients with familial neurodegenerative disorders, including schizophrenia and encephalopathy, has uncovered a strong relationship between ATAD1 mutations and these diseases [19,22]. Our study further reveals that the depletion of ATAD1 not only impairs neuronal development but also critically affects both synapse formation and synaptic transmission. Next, we determined whether ATAD1-mediated mitochondrial fission contributes to neuronal damage. Our previous research has confirmed that Msp1, an ATAD1 homolog in yeast, regulates mitochondrial fission independently of its hydrolase activity [24]. Consistent with this, our findings indicate that the regulation of mitochondrial fission by ATAD1 in neurons also does not depend on the hydrolysis site of ATAD1. However, the hydrolytic site of ATAD1 is crucial for spine formation in hippocampal neurons. This strongly suggests that the modulation of synapse formation by ATAD1 depends on its regulation on mitochondrial function.

Several aspects of mitochondrial morphology and functions have been shown to play a crucial role in modulating synaptic functions [35]. One study showed that mitochondrial fragmentation resulting from the knockdown of OPA1 disrupts synapse maturation [36]. Knocking down DRP1, a key fission mediator, decreases ATP levels and oxygen consumption in hippocampal neurons, which, in turn, impairs synaptic function and leads to deficits in learning and memory [37]. However, in our study, we found that the neuronal deficits associated with ATAD1 loss is not rescued by the restoration of mitochondrial morphology with a mutant ATAD1 without ATP hydrolysis. This suggests that the mitochondria-mediated modulation on synapses is not dependent on the mitochondrial morphology per se but on related changes in mitochondrial functions. While low levels of ROS can serve signaling functions including stabilizing long-term potentiation (LTP) at excitatory synapse [38] and regulating the strength of synaptic transmission at inhibitory synapses [39], a sustained elevation of ROS is often detrimental [40,41]. We found that ATAD1 deficiency results in a significant increase in the mitochondrial ROS level. This sustained high level of ROS can lead to the increased oxidative damage of mitochondrial proteins and mitochondrial DNA, exacerbating mitochondrial dysfunction [42]. Meanwhile, mitochondria serve as the energy source required for neuronal electrical activity, neurite outgrowth, neurotransmission, synaptogenesis, and synaptic plasticity [43,44,45]. The alterations in both mitochondrial morphology and function may all converge to the changes in ATP production [46,47,48]. Indeed, we found that ATAD1 deficiency results in a strong reduction of mitochondrial ATP level in cultured neurons. In addition to mitochondrial dynamics, several aspects of mitochondrial calcium have been shown to play a crucial role in modulating synaptic functions. Mitochondria effectively buffer Ca^2+^ influx during neuronal excitation and subsequently release accumulated Ca^2+^ into the cytosol [49]. Consequently, mitochondria exhibit a profound capacity to regulate and modulate Ca^2+^-dependent neuronal functions, including excitability, neurotransmission [50]. The reduction in the amplitude of mitochondrial Ca^2+^ activities upon ATAD1 deficiency in our study suggest that the absence of ATAD1 disrupts mitochondrial matrix calcium homeostasis. Since ATAD1 plays a key role in mitochondrial quality control by removing mislocalized tail-anchored (TA) proteins from the mitochondrial outer membrane [31,33], the deficiency in this process may contribute to the mitochondrial dysfunctions we have observed.

Perturbations of mitochondrial function have been recognized as pivotal contributors to the pathogenesis of neurological disorders and neurodegenerative diseases [51,52]. Seizures frequently represent a primary manifestation of neurological disorders resulting from pathogenic mutations in 169 identified genes that impact mitochondrial function [53]. The observed impairment of mitochondrial function has a potential direct link to the increased neuronal excitability that leads to epileptiform activity, which includes reduced ATP levels, alterations in neuronal calcium homeostasis, and modifications of ion channels and neurotransmitter transporters induced by reactive oxygen species [54]. In animal models related to Alzheimer’s disease, abnormal mitochondrial function is a common cellular feature. Inhibiting Drp1 has been shown alleviate the loss of mitochondrial membrane potential, reduce ROS production, restore ATP levels, and alleviate synaptic depression in neurons treated with Aβ [55]. In Huntington’s disease, as observed in Alzheimer’s disease, mitochondrial functions are profoundly disrupted. Post-mortem brain tissue from Huntington’s disease (HD) patients and striatal cells from mice with mutant huntingtin knock-in exhibit a pathological-dependent reduction in mitochondrial numbers, and both mitochondrial respiration and ATP production are markedly impaired [56]. Neurons affected by Parkinson’s disease often exhibit the reduced activity of oxidative phosphorylation complexes and/or elevated levels of mitochondrial DNA loss [57]. Biochemical and genetic studies indicate that the products of two mutated genes, PINK1 and Parkin, associated with autosomal recessive Parkinson’s disease, typically collaborate in the same pathway to govern the mitochondrial quality control system, thereby influencing the onset and progression of Parkinson’s disease [58]. To date, ATAD1 has not been directly linked to the onset of neurodegenerative diseases. However, it has been identified as a gene associated with the neurodevelopmental disorder stiff baby syndrome [19], and its role in promoting neuronal survival following injury, such as stroke [20], suggests that ATAD1 may contribute to the pathogenesis of neurological disorders. Our findings suggest that ATAD1 deficiency may represent a novel pathological mechanism underlying this rare disease by disrupting mitochondrial function, thereby impairing neuronal development and synaptic transmission. This further emphasizes the importance of understanding the role of ATAD1 in mitochondrial modulation and its potential implications for cognitive impairments in neurological diseases.

Our study offers important insights into the role of ATAD1 in mitochondrial function and synaptic activity within hippocampal neurons. However, we acknowledge several limitations that should be considered. First, our research was conducted primarily on cultured hippocampal neurons, which may not fully capture the complexity of ATAD1’s role in vivo, especially regarding intact brain circuits and various neuronal subtypes. Future studies utilizing in vivo models will be essential to validate our findings and investigate potential region-specific effects of ATAD1 deficiency. Second, while we established that the ATP hydrolysis activity of ATAD1 is critical for alleviating synaptic deficits, the exact molecular pathways through which ATAD1 influences mitochondrial function and synaptic transmission remain unclear. Subsequent research should focus on elucidating the downstream signaling mechanisms affected by ATAD1 to provide a more comprehensive understanding of its cellular roles. In summary, our findings underscore the significance of ATAD1 in mitochondrial dynamics and neuronal function, but further investigation is necessary to clarify its broader implications in both physiological and pathological contexts.

## 4. Materials and Methods

### 4.1. Primary Dissociated Hippocampal Neuronal Cultures

For hippocampal neuron culture experiments, CD^®^(SD) IGS rats were obtained from Charles River. Rat experiments were approved by the University of Science and Technology of China of the Animal Care and Use Committee requirements (approval no. USTCACUC26120223022). The housing conditions for rats involved an inverted 12 h light–dark cycle, maintaining a stable temperature of 22 ± 2 °C, and rats were provided ad libitum access to food and water. Hippocampi were dissected from embryonic day-18 CD^®^(SD) IGS rats. Briefly, hippocampi were isolated in cold Hanks’ balanced salt solution (HBSS) without Ca^2+^ and Mg^2+^ (Thermo Fisher, Waltham, MA, USA), followed by a digestion process employing 0.25% trypsin (Sigma-Aldrich, St. Louis, MO, USA) at 37 °C for 15 min. The dissociated neurons were plated on 50k cells/18 mm coverslips in 12-well plates, which were coated overnight with 0.01% poly-L-lysine (Sigma-Aldrich, Cat# P1274) at 37 °C. Neurons were maintained in neuronal medium supplemented with 2% B27, 1% 100× GlutaMax, 1% 1 M NaCl at a temperature of 37 °C within a 5% CO_2_ incubator.

### 4.2. Plasmids and Transfection

DNA plasmids for mito-DsRed, mito-rGECO and Mito-BFP were kindly gifted by Dr. Thomas A Blanpied (University of Maryland School of Medicine). We constructed the plasmid shATAD1-GFP (sequence: gcgtcagggaatatgtcaatt) was uesd PLL5.0 as the vector, a stem-loop structure was inserted downstream of the U6 promoter through digestion with restriction enzymes (Thermo Fisher) and ligation with T4 ligase enzymes (Sangon Biotech, Shanghai, China). We constructed the plasmids ATAD1-GFP, shATAD1-mcherry, ATAD1-mcherry, E193Q-mcherry by homologous recombination using ClonExpress II One Step Cloning Kit (Vazymebiotech, C112, Nanjing, China). Neurons were transfected with 0.5–1 µg plasmids at approximately 10–11 DIV using a calcium phosphate transfection kit (Takara, Cat# 631312, San Jose, CA, USA), except for developmental experiments, neurons were transfected at 3 DIV.

### 4.3. Immunocytochemistry and Immunohistochemistry

Cultured rat hippocampal neurons were fixed with 4% paraformaldehyde (PFA) for 10 min and subsequently washed with PBS containing 100 mM glycine three times for 5 min each. Neurons were permeabilized and blocked with a solution composed of 0.3% Triton X-100, 3% BSA, and 5% donkey serum in PBS containing glycine for 1 h. The neurons were then incubated with primary antibodies that were diluted in a buffer consisting of 0.3% Triton X-100, 3% BSA, and 5% donkey serum in PBS containing glycine solution at room temperature for 3 h, followed by three 5 min washes. The primary antibodies employed included mouse anti-PSD95 (1:200, NeuroMab, 75-028, Davis, CA, USA), rabbit anti-PSD95 (1:500, Cell Signaling Technology, 3409S, Danvers, MA, USA), Guinea pig anti-GABA_A_R (1:200, SYSY, Goettingen, Germany, 224004) and mouse anti-ATAD1 (1:500, Sigma-Aldrich, Q2444401) and rabbit anti-ATAD1 (1:500, this paper, N/A). Finally, the neurons were incubated at room temperature for 1 h with secondary antibodies that were diluted in PBS containing glycine solution and washed three times for 5 min each. The secondary antibodies included anti-rabbit Alexa Fluor 647 (1:200, Jackson ImmunoResearch, 711-605-152), donkey anti-Guinea pig Alexa Fluor 647 (1:200, Jackson ImmunoResearch, 706-605-148, West Grove, PA, USA), donkey anti-mouse Alexa Fluor 647 (1:200, Jackson ImmunoResearch, 715-605-151), donkey anti-rabbit Alexa Fluor 488 (1:200, Jackson ImmunoResearch, 711-545-152), donkey anti-mouse Alexa Fluor 488 (1:200, Jackson ImmunoResearch, 715-545-151) and donkey anti-mouse Cy3 (1:200, Jackson ImmunoResearch, 715-165-151). The neurons were mounted with antifade mounting medium.

### 4.4. Live Cell Imaging and Ca^2+^ Imaging

For dendritic complexity analysis, neurons were transfected at DIV3 and imaged at DIV7 or DIV10 in imaging buffer with a pH of 7.4, consisting of 120 mM NaCl, 3 mM KCl, 10 mM glucose, 10 mM HEPES, 2 mM MgCl_2_ and 2 mM CaCl_2_. To label the mitochondria, neurons were incubated in the imaging buffer at a final concentration of 0.3 µM MitoTracker (Thermo Fisher, M7152) for 30 min. For dendritic spine morphology analysis, neurons were transfected at DIV10-11 and imaged at DIV18-20 in imaging buffer. For analysis of mitochondrial Ca^2+^ amplitude or cytoplasmic Ca^2+^ amplitude, neurons were transfected with genetically encoded a red fluorescent mitochondrial Ca^2+^ indicators (mito-rGECO). Images were acquired using a W1 spinning disc confocal microscope (Ti2E, NIKON, Tokyo, Japan) with a 100× oil objective (1.45 NA, NIKON) or a spinning disk confocal CSU-10.3 with a Zyla5.5 sCMOS camera (Andor, Belfast, Northern Ireland) on an Olympus IX-71 inverted microscope (100× 1.49 NA oil objective, Olympus, Tokyo, Japan) and analyzed by Fiji ImageJ 1.54. For neuronal cultures, all quantifications were performed on the secondary dendrites of transfected neurons. A region of interest (ROI) was chosen based on the imaging criteria, such as fluorescent signal quality and background level. The mitochondrial density and the PSD-95 density was calculated as the total number of mitochondrial/PSD95 puncta in all segments of secondary dendrites within the ROI, divided by the total length of the branches. Typically, one representative ROI was imaged per neuron, and the data were averaged across neurons/ROIs.

### 4.5. Whole-Cell Patch-Clamp and Field EPSP Recording

Cultured rat hippocampal neurons were visualized with 40× water-immersion objective on an upright microscope (BX51WI, Olympus, Tokyo, Japan), with interference contrast (IR/DIC) and an infrared camera connected to the video monitor. For whole-cell patch-clamp, patch pipettes were pulled to a resistance of 6–8 MΩ by horizontal pipette puller (P-97, Sutter Instruments, Novato, CA, USA). The patch pipettes were filled with the internal solution composed of 130 mM K-gluconate, 5 mM KCl, 2 mM MgCl_2_, 10 mM HEPES, 4 mM Mg-ATP, 0.3 mM Na-GTP and 0.6 mM EGTA. The neurons were recorded miniature excitatory postsynaptic current (mEPSC) in imaging buffer containing 50 μM picrotoxin (PTX), 1 μM tetrodotoxin (TTX) and 50 μM 2-Amino-5-phosphonovaleric acid (APV) at a holding potential of −70 mV. All recording data were obtained using MultiClamp 700B amplifiers (Molecular Devices, San Jose, CA, USA), filtered with a 2.8 kHz low-pass filter, digitized at 10 kHz with a Digidata 1440A Data Acquisition System and analyzed using Clampfit 10.7 software (Molecular Devices, Sunnyvale, San Jose, CA, USA) and MiniAnal software 6.0.3.

### 4.6. Mitochondrial Function Experiment

For neuronal cultures, to assess mitochondrial membrane potential, neurons were subjected to incubate with 175 μM 3,3′-dihexyloxacarbocyanine iodide (DiOC6, Thermo Fisher, D273) in the imaging buffer for 30 min at 37 °C. To determine of ATP levels, neurons were stained with 5 μM ATP-Red1 (MCE, Shanghai, China, HY-U00451) for 30 min in the imaging buffer at 37 °C. To determine oxygen species (ROS), neurons were incubated with 10 μM Dihydrorhodamine 123 (DHR123, MKBio, Shanghai, China, MX-4455) in the imaging buffer for 30 min at 37 °C.

### 4.7. Quantification and Statistical Analysis

Fiji ImageJ was used for the analysis of the image signal data generated by confocal microscopy. GraphPad Prism 9, Origin 2019, MATLAB 2018b and Adobe Illustrator CC 2018 were used for the statistical analyses and graphing. Offline analysis of the data obtained from electrophysiological recordings was conducted using Clampfit software version 10.7 and MiniAnal 6.0.3 software. For two-group comparisons, we employed either the Student’s t-test or the Mann–Whitney U test, depending on the data distribution. The Student’s *t*-test was applied when the data followed a normal distribution, whereas the Mann–Whitney U test was used when the assumption of normality was not met. For three-group comparisons, we utilized one-way ANOVA, Welch ANOVA, Kruskal–Wallis test, along with post hoc Dunnett’s test as appropriate. One-way ANOVA was applied when the data met the assumptions of normal distribution and equal variances. Welch’s ANOVA was employed when the data followed a normal distribution but had unequal variances, and the Kruskal–Wallis test was employed when the data did not meet the normality assumption. Additionally, two-way ANOVA with Dunnett’s post hoc test was applied to the Sholl analysis data. The K-S test was used for the frequency distribution graph. Significance levels are displayed as * *p* < 0.05, ** *p* < 0.01, *** *p* < 0.001, **** *p* < 0.0001 and not significant (ns.), and the results are expressed as the mean ± s.e.m.

## Data Availability

All data supporting the findings of this study will be shared by the lead author Ai-Hui Tang upon request. This paper does not report original code. All data needed to evaluate the conclusions in the paper are present in the paper and/or the Supplementary Materials.

## Supplementary Materials

The supporting information can be downloaded at: https://www.mdpi.com/article/10.3390/ijms26010044/s1.
