# Supplementary material for: ATAD1 Regulates Neuronal Development and Synapse Formation Through Tuning Mitochondrial Function
Source: Int J Mol Sci. 2024 Dec 24;26(1):44. doi: 10.3390/ijms26010044 (PMC11719905; doi:10.3390/ijms26010044)
Supplement: Supplementary file 1 [file ijms-26-00044-s001.zip › ijms-3324364-supplementary.pdf]

Supplementary Materials for

ATAD1 regulates neuronal development and synapse formation through tuning mitochondrial function

Hao-Hao Yan *et al.*

*Corresponding author: Jia-Hui Chen, Email: chenjh95@ustc.edu.cn

Ai-Hui Tang, Email: tangah@ustc.edu.cn

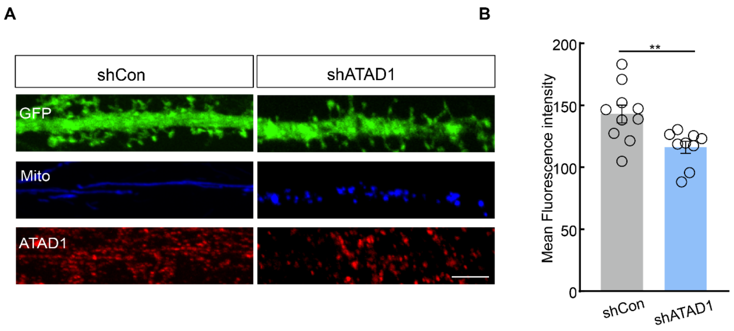

Figure S1. The levels of ATAD1 protein were reduced in neurons transfected with shATAD1.

(**A**) Representative images of ATAD1 staining (red) in neurons transfected with shATAD1 plasmids. Scale bar 5 μm.

(**B**) Quantification of ATAD1 intensity show the efficiency of neuronal knockdown of the ATAD1 protein (n = 9-10 cells/3 culture, ** $p = 0.0057$).

Significance was assessed by Mann–Whitney tests (**B**). All data are presented as the mean ± s.e.m. ** $p < 0.01$.

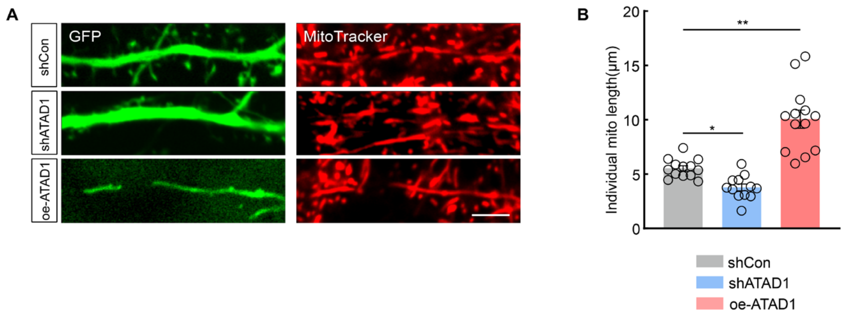

Figure S2. ATAD1 deficiency results in mitochondrial fragmentation in neurons.

(**A**) Representative images of MitoTracker (red) in shCon-expressing, shATAD1-expressing or oe-ATAD1-expressing neurons. Scale bar 5 µm.

(**B**) Quantification of individual mitochondrial length (n = 12-13 cells/3 cultures, $p < 0.0001$).

Significance was assessed by Kruskal-Wallis test (**B**). All data are presented as the mean ± s.e.m. * $p < 0.05$, ** $p < 0.01$.

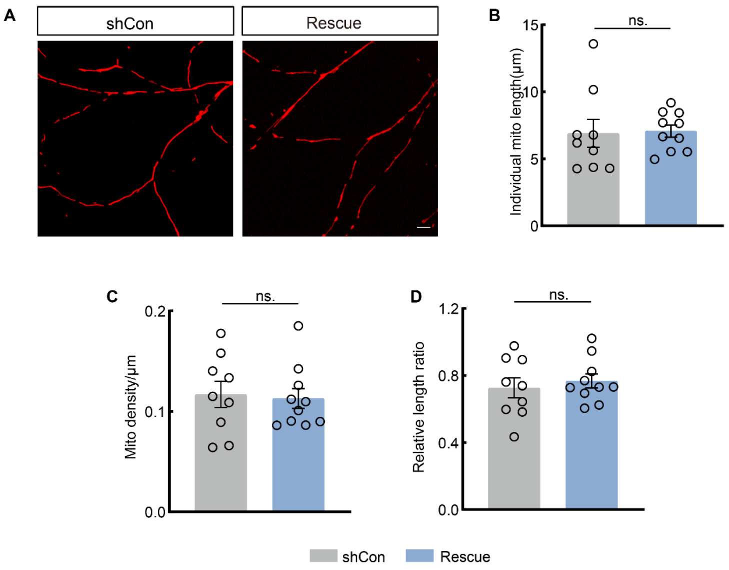

Figure S3. Overexpressing ATAD1 rescues mitochondrial morphology in cultured hippocampal neurons.

(**A**) Representative images of mitochondrial morphology in cultured hippocampal neurons expressing shCon, or coexpressing shATAD1 and oe-ATAD1. Scale bar 5 μm.

(**B**) Quantification of individual mitochondrial length along dendrites (n = 9-10 cells/3 culture, P = 0.4967).

(**C**) Quantification of the mitochondrial density (n = 9-10 cells /3 culture, P = 0.8421).

(**D**) Quantification of the relative length ratio (n = 9-10 cells /3 culture, P = 0.5668).

Significance was assessed by Student's t tests (**D**), or Mann–Whitney tests (**B** and **C**). All data are presented as the mean ± s.e.m. ns., not significant.

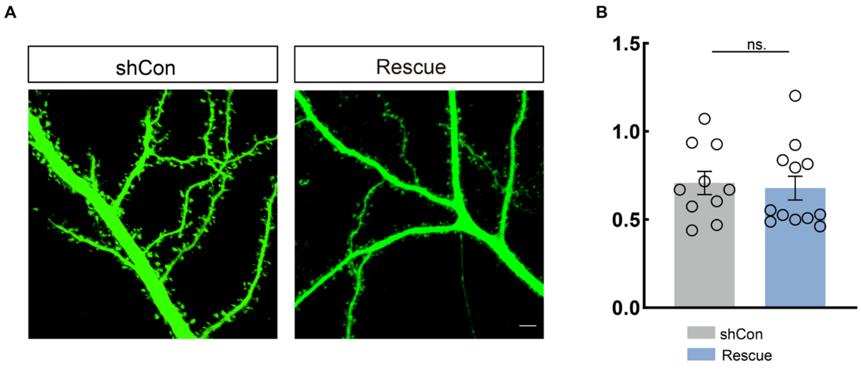

Figure S4. Overexpressing ATAD1 rescues spine density in cultured hippocampal neurons.

(**A**) Representative images of neuron morphology and dendritic spines in rat hippocampal neurons expressing shCon, or coexpressing shATAD1 and oe-ATAD1. Scale bar 5 μm.

(**B**) Quantification of neuronal dendritic spines (*n* = 10-12 cells/3 cultures, *P* = 0.5824).

Significance was assessed by Mann–Whitney tests (**B**). All data are presented as the mean ± s.e.m. not significant.

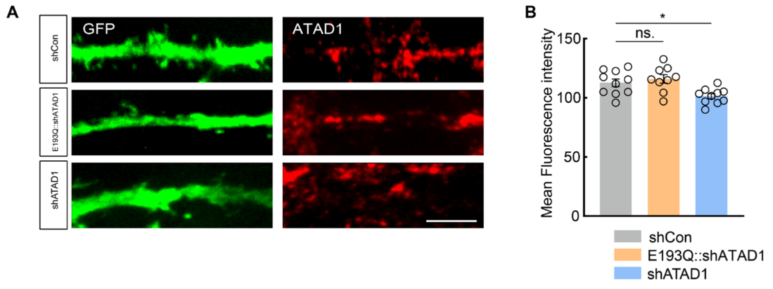

Figure S5. The levels of ATAD1 protein in neurons transfected with ATP hydrolysis-deficient mutant.

(**A**) Representative images of ATAD1 staining (red) in shCon-expressing, E193Q::shATAD1-expressing (E193Q and shATAD1-coexpressing) or shATAD1-expressing neurons. Scale bar 5 μm.

(**B**) Quantification of ATAD1 intensity (n = 9-10 cells/3 culture, P = 0.0048).

Significance was assessed by one-way ANOVA measures (**B**). All data are presented as the mean ± s.e.m. * $p < 0.05$, ns., not significant.

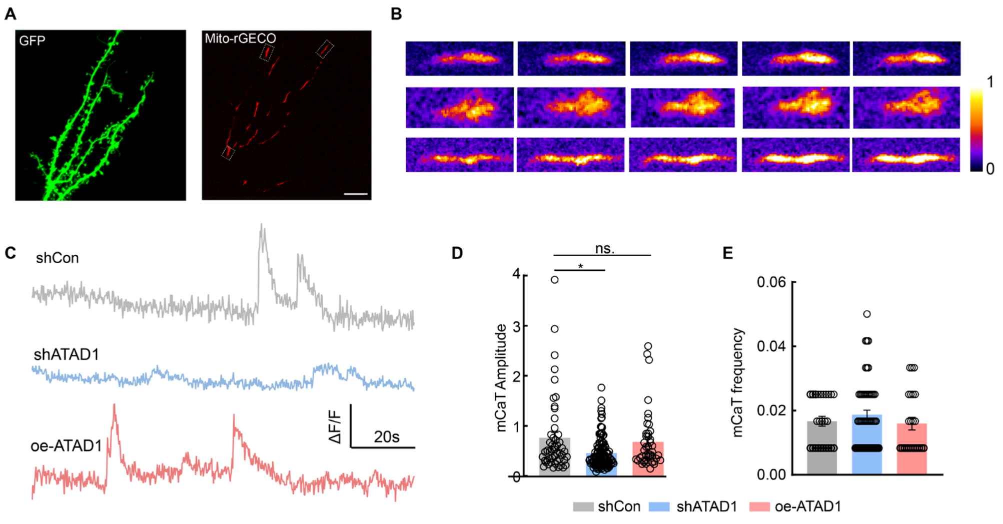

Figure S6. ATAD1 deficiency disrupts mitochondrial matrix Ca^{2+} homeostasis in neuorns.

(**A**) Representative images of cultured rat hippocampal neuron expressing GFP and Mito-rGECO. Scale bar 10 μm.

(**B**) Representative Mito-rGECO heatmap of dendritic mitochondria.

(**C**) Representative Mito-rGECO traces in shCon-expressing, shATAD1-expressing or oe-ATAD1-expressing cultured rat hippocampal neurons neurons.

(**D-E**) Quantification of mCaT amplitude (**D**) and frequency (**E**) in shCon-expressing, shATAD1-expressing or oe-ATAD1-expressing cultured rat hippocampal neurons (n = 23-54 mitochondria/3 cultures, **D**, $P = 0.0033$, **E**, $P = 0.5507$).

Significance was assessed by Kruskal-Wallis tests (**D** and **E**). All data are presented as the mean ± s.e.m. $*P < 0.05$, ns., not significant.
